# Operationalization of Intrinsic Capacity in Older People and Its Association With Subsequent Disability, Hospital Admission and Mortality: Results From The English Longitudinal Study of Ageing

**DOI:** 10.1093/gerona/glac250

**Published:** 2022-12-13

**Authors:** Charlotte L Campbell, Dorina Cadar, Anne McMunn, Paola Zaninotto

**Affiliations:** Department of Epidemiology and Public Health, University College London and CLOSER, Social Research Institute, University College London, London, UK; Centre for Dementia Studies, Brighton and Sussex Medical School and Department of Behavioural Science and Health, University College London, London, UK; Department of Epidemiology and Public Health, University College London, London, UK; Department of Epidemiology and Public Health, University College London, London, UK

**Keywords:** Dependence, Index, Survival

## Abstract

**Background:**

Intrinsic capacity (IC) is a new concept in the healthy aging field and has many operationalized definitions. In this study, we operationalized IC using item response theory in the English Longitudinal Study of Ageing (ELSA) and tested the predictive value of the scale using a subsequent functional ability, mortality, and hospital admission.

**Methods:**

IC was measured at baseline (2004, Wave 2) using 14 dichotomous indicators: word recall, orientation in time, balance, chair rises, walking speed, upper mobility, lower mobility, eyesight, hearing, grip strength, body mass index, waist circumference, depressive symptoms, and life satisfaction. A 2-parameter item response theory model was used to generate a scale of IC at baseline. Logistic regression was used for the prediction of subsequent difficulties, measured by difficulties with ≥1 activities of daily living (ADLs) and ≥1 instrumental activities of daily living (IADLs) at 4 and 8 years after baseline. Competing risk and Cox regressions were employed to test the prediction of hospital admission and mortality, respectively, over a 14-year follow-up.

**Results:**

IC scores were generated for 4 545 individuals aged on average 70.8 years (standard deviation [*SD*] 7.93). Better baseline IC scores were associated with reduced risk of subsequent difficulties with ADLs and IADLs, hospital admission (subdistribution hazard ratios [SHR] = 0.99, 95% confidence interval [CI] 0.98–0.99), and mortality (hazard ratios [HR] = 0.98, 95% CI 0.98–0.99), when adjusted for sociodemographic and health-related covariates.

**Conclusion:**

These results suggest the utility of this IC score as a measure of risk for future adverse outcomes in older people, potentially above that indicated by other sociodemographic and health-related factors.

With population aging becoming commonplace across many areas of the world, the health requirements of many societies are shifting. As such, the World Health Organization (WHO) has recently outlined a healthy aging framework to guide public health action (1). Under this framework, healthy aging is defined as maintaining a level of functional ability that enables well-being into older age, with functional ability comprised of an individual’s intrinsic capacity (IC), their environment, and the interaction between the 2. IC is described as the “composite of all the physical and mental capacities of an individual” (1, p. 28) and has been largely adopted as the measurable part of the WHO framework, with the idea that monitoring IC over time could alert to individuals experiencing declines in capacity ahead of a negative outcome. IC is composed of 5 domains of capacity (2): cognition, locomotion (mobility and muscular function), sensory (vision and hearing),  vitality (energy balance and nutrition), and psychological mood. These domains were identified as the body and mind functions associated with a higher risk of adverse health outcomes such as disability, frailty, care dependency, and mortality (2). Although there is no consensus on a standard index of IC (3), the indicators used generally follow this 2-domain structure. There is also no agreement on the generation of a total score of IC, with many models only producing domain-specific scores (3). In an early model of IC, Beard et al. used confirmatory factor analysis (CFA) to generate a bifactor model of IC and extract a score for the general IC factor (4), while others have used composite *z*-scores (5,6) and summing of item scores or impairments (7,8).

That there is no agreed method of generating an IC score may be due to the different limitations of each of these methods. The sum scores of indicators are simple and easy to understand, but without weighting, they assume that each indicator is equally important to IC. They are also critically reliant on the indicators chosen for each domain, where there is again no consensus. On the other hand, factor scores capture the different weighting of the items in the total score but, being a data-driven approach, are dependent on the data distribution and sample used to derive them—a problem also for *z*-scores.

Item response theory (IRT) is another data-driven method used to assess latent factors in a similar way to CFA (9). IRT is increasingly used in a wide range of disciplines and has applications in patient-reported outcomes and clinical assessment (10). The IRT method links item responses to a latent trait, assuming that the respondent’s natural position on the trait influences their probability of a certain response category on the item. IRT can provide information about how items capture information across the range of a latent construct and how well the scale operates at all levels of the latent trait (11). As a data-driven method, IRT suffers from some of the same limitations of CFA but its focus on items means it is more suited to examining individual item characteristics or estimating scores for respondents, while CFA is more appropriate when focusing on the structural makeup of a scale. IRT has been successfully applied to constructs of healthy aging by the Ageing Trajectories of Health-Longitudinal Opportunities and Synergies (ATHLOS) consortium, which generated a total factor score representing a healthy aging scale across 16 cohorts across 38 countries (12). The healthy aging score was then found to correspond well with functional health status and was a predictor of mortality. Salinas-Rodríguez et al. (13) used IRT to generate an IC score over 3-time points, finding those with a slightly increasing trajectory showed higher quality-of-life and lower disability. However, an IRT model of IC is yet to be explored using English data.

Therefore, the aim of this study was to operationalize IC using IRT methodology in a large national sample of older individuals taking part in the English Longitudinal Study of Ageing (ELSA). This model is intended to be simple enough to be replicated in other studies of aging and allow for the possibility of modeling IC over time. The predictive validity of the measure is assessed through its association with subsequent functional ability, mortality, and hospital admissions.

## Method

### Sample

We included 5 343 members aged ≥60 of the ELSA (14), a large, ongoing, nationally representative prospective cohort study of older adults living in private households in England. All ELSA participants provided written consent prior to the study, and ethical approval was granted by the London MultiCentre Research Ethics Committee. Data are made available through the UK Data Service.

### Intrinsic Capacity

IC was measured in those aged ≥60 using 14 indicators covering the 5 domains of capacity: cognition, locomotion, sensory function, vitality, and psychological well-being. All indicators were chosen due to their association with functional ability decline and adverse outcomes. More details can be found in Supplementary Table 1.

The cognition domain was measured with a word recall test and orientation in time, while locomotion was measured with the Short Physical Performance Battery (15) (balance tests, chair rises, and walking speed), upper mobility, and lower mobility. The sensory domain included self-rated eyesight and hearing, while vitality was measured with grip strength, body mass index, and waist circumference. Finally, the psychological domain included the Center for Epidemiological Studies Depression scale (16) and the Satisfaction with Life Scale (17).

### Outcome Measures

Subsequent functional ability was measured with activities of daily living (ADLs; 6 activities) and instrumental activities of daily living (IADLs; 7 activities), with each binarised into “no difficulties” and “1 or more difficulties.” ADLs and IADLs were measured at wave 2 (2004/2005) to establish baseline status, and then at wave 4 (2008–2009) and at wave 6 (2012–2013). Mortality up to April 2018 was determined from linked mortality register data. Hospital admissions up to January 2018 were gathered using electronic health records and linked to survey members.

### Covariates

Baseline covariates included age, sex, marital status, highest educational qualification, total net wealth, occupation, alcohol consumption, smoking, level of physical activity, number of health conditions, and self-rated health (see Supplementary Table 2). These socioeconomic and health-related factors were chosen as they are known to be associated with health outcomes in older people (18) and may confound the current analysis.

### Statistical Analysis

The IC score was generated using a 2-parameter logistic IRT model where the probability of experiencing “no difficulty” on an indicator was modeled as a function of 2 item parameters (discrimination and difficulty) and a person parameter. Full information maximum likelihood (FIML) was used to estimate the item and person parameters when values were missing, while expected a posteriori estimation was used to calculate a factor score for each individual. FIML is one of the most commonly used approaches to deal with missing data, especially for IRT, and has been found to deal with missing data in IRT models with higher accuracy than other zero replacement and multiple imputation methods (19).

Model fit was assessed with the root mean square error of approximation (RMSEA), the comparative fit index (CFI), and the Tucker–Lewis index (TLI). To assess the model fit, missing values on the IC indicators were imputed 10 times based on the latent trait value and item parameters of the estimated IRT model; the average model fit statistic from the imputations was reported. The resulting factor scores from the IRT model were extracted and standardized to have a mean of 50 and a standard deviation (*SD*) of 10.

Linear regression was used to test the association between the IC scores and covariates (at baseline, 2004). Logistic regression was used to test the association between the IC score and the binary I/ADLs outcomes (at 4- and 8-year follow-up), adjusted for baseline I/ADLs status and covariates. Cox proportional hazard models with hazard ratios (HR) were performed to test the association between the IC scores and mortality during the 14-year follow-up. Competing-risk regression analysis with subdistribution hazard ratios (SHR) was used for the association between IC scores and hospital admission, with death as a competing risk, using a version of the Fine and Gray method (20). Censoring was set at April and January 2018 for hospital admission and mortality, respectively. Both the mortality and hospital admission fully-adjusted analyses included all covariates outlined previously.

The main sample included 4 545 individuals who had an IC score and no missing data on covariates at baseline and gave consent for data linkage with hospital and mortality records. A total of 3 055 individuals also had information on ADLs and IADLs at wave 4, while 2 348 had this information at wave 6. Among the 4 545 individuals in the main sample, 4 489 were included in the competing risk analysis as they experienced admission to the hospital or death or reached the censoring point.

All analyses were carried out in Stata/SE v16.1 (21), and R (22), using the package *mirt* (23).

## Results

An IC score was generated for 4 545 cohort members, 55% were female, and the overall sample means age was 70.8 years (*SD* 7.93). The IRT model converged successfully with a good fit (RMSEA = 0.06, TLI = 0.90, CFI = 0.91). The parameters revealed that locomotion items had the highest discrimination, while orientation and waist circumference had the lowest (Supplementary Table 3). Orientation was found to have the lowest difficulty, while waist circumference had the highest. The IC factor scores ranged from 20 to 66, with a mean of 50.7 (*SD* 9.80) and were left-skewed.

**Figure 1.**
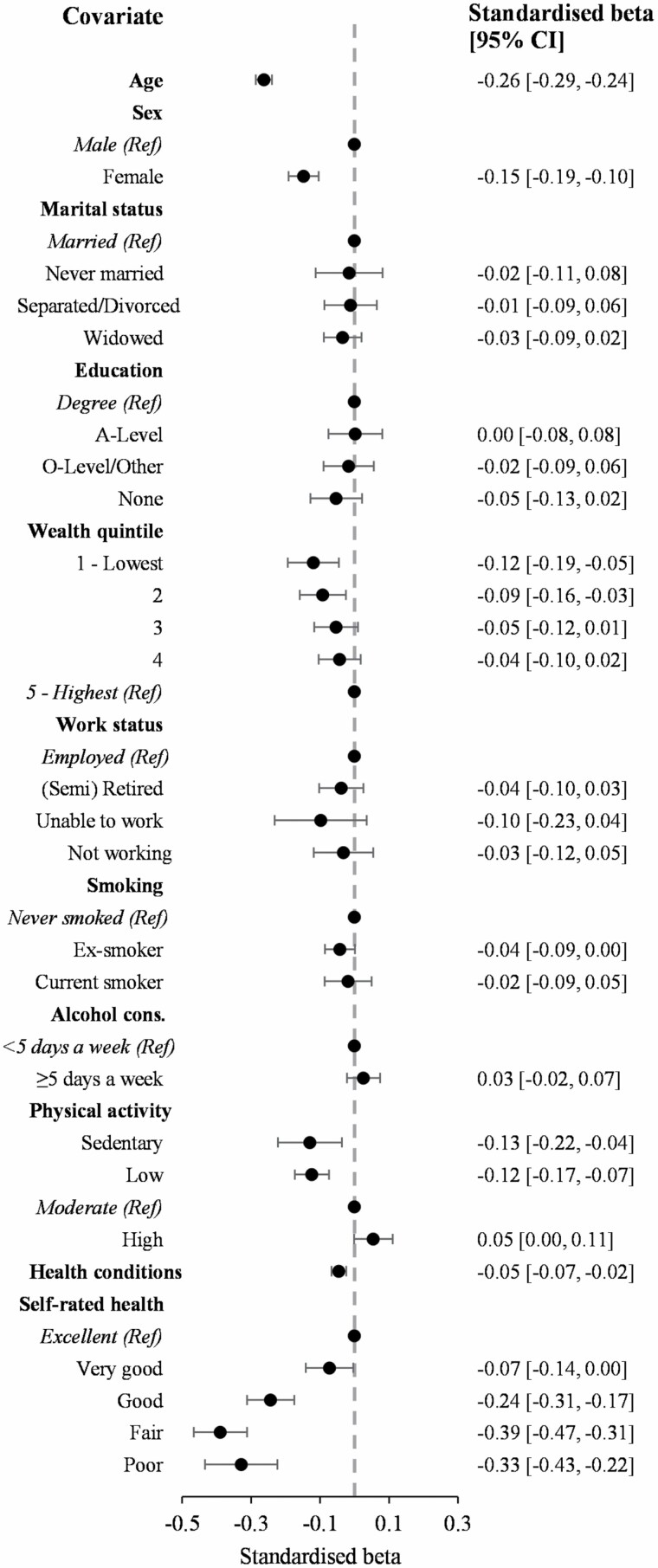
Forest plot for the linear regression between intrinsic capacity scores and sociodemographic and health-related covariates at baseline (*N* = 4 545). Standardized beta coefficients are presented. CI = confidence interval.

In the fully-adjusted linear regression model, a significant association was found between IC scores and most covariates (Figure 1, Supplementary Table 4). Lower IC scores were associated with older age (β = −0.32, 95% confidence interval [CI] −0.35 to −0.30), women (β = −2.90, 95% CI −3.33 to −2.48), and those in lower wealth quintiles (lowest quintile β = −3.16, 95% CI −3.88 to −2.43) and not in employment (retired β = −0.86, 95% CI −1.49 to −0.23), with lower physical activity levels (sedentary β = −5.29, 95% CI −6.20 to −4.39), more health conditions (β = −0.36, 95% CI −0.53 to −0.19), and lower self-ratings of health (poor β = −12.49, 95% CI −13.52 to −11.46).

**Figure 2.**
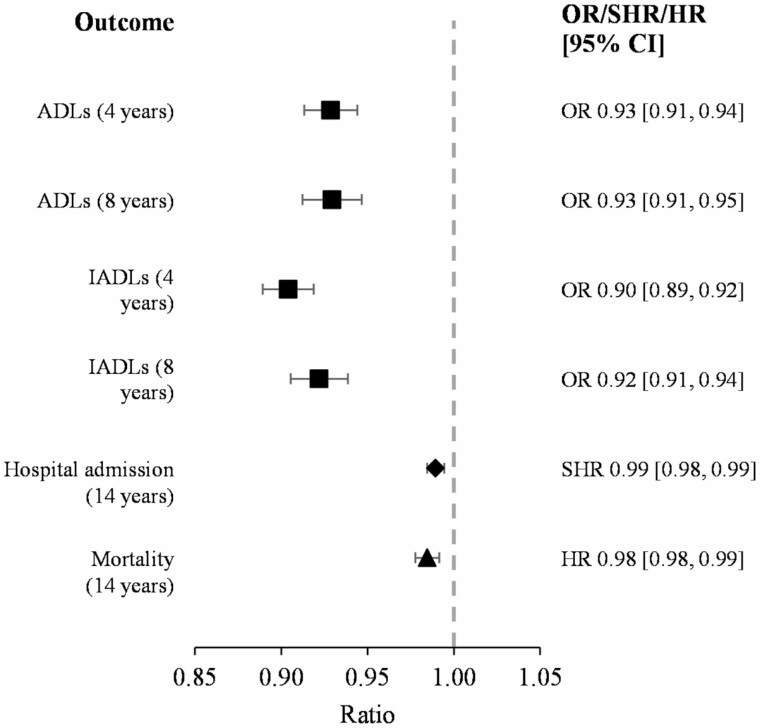
Forest plot for the association between baseline intrinsic capacity scores and future outcomes: ADLs and IADLs at 4 years later (*N* = 3 055) and 8 years later (*N* = 2 348), and hospital admission (*N* = 4 489) and mortality (*N* = 4 545) during the 14 years follow-up. Odds ratio (OR, ■), subdistribution hazard ratio (SHR, ♦), or hazard ratio (HR, ▲) are presented depending on the analysis. ADL = activities of daily living; CI = confidence interval; IADL = instrumental activities of daily living.

Logistic regression analyses revealed that baseline IC score was significantly negatively associated with experiencing one or more difficulties with ADLs and IADLs at waves 4 and 6 when adjusting for previous difficulties with ADL/IADLs and covariates (Figure 2, Supplementary Table 5). Those with a higher IC score at baseline were 7%–10% less likely to experience difficulties with ADLs and IADLs 4 and 8 years later.

Among the 4 545 individuals in the mortality analytical sample, 40.2% died within the follow-up period. Higher IC scores were significantly associated with a lower risk of mortality (Figure 2, Supplementary Table 6), with a 1-unit increase in IC score decreasing the probability of death within 14 years by 2% (HR = 0.98, 95% CI 0.98–0.99) in the fully adjusted analysis.

The follow-up time to the first hospital admission or competing event ranged between 0.08 and 13.55 years, with a mean of 3.85 years for those who were admitted to the hospital, 6.64 years for those who died, and 13.17 years for those who experienced neither event. In total, 3 784 admissions to the hospital were recorded, and 184 deaths were considered a competing event. Competing risk analysis revealed that a higher IC score was associated with a reduced risk of hospital admission, even when adjusted for covariates (Figure 2, Supplementary Table 6). In fully-adjusted analyses, a 1-unit increase in IC score was associated with a 1% reduction (SHR = 0.99, 95% CI 0.98–0.99) in the probability of hospital admission within 14 years. Sensitivity analyses using Cox proportional hazard models revealed similar patterns (Supplementary Table 7). Tests of the proportional hazards assumption revealed no violations.

## Discussion

This study has computed an IC score in a representative sample of adults aged ≥60 years from private households in England. The IC score was associated with sociodemographic and health-related factors. Importantly, the IC score was found to significantly predict subsequent functional ability, hospital admission, and mortality over a period of up to 18 years, even when adjusted for other health conditions. A 1 unit increase in IC was found to reduce the risk of ADL/IADL difficulties by 7%–9%, mortality by 2%, and hospital admission by 1%. As a standardized factor score of a latent factor, 1 unit of IC does not have any inherent meaning; however, to understand what this means in terms of effect, a 2-unit reduction in IC score would be equivalent to having a diagnosis of high cholesterol or arthritis.

The findings that the IC score has the predictive ability for objective health outcomes are consistent with previous similar studies. The ATHLOS consortium found their healthy aging index, also generated with an IRT model, was associated with sociodemographic and health factors as well as predicted mortality, although the magnitude of this prediction was not tested (12). A study of community-dwelling older adults aged ≥70 years in the United States found a 1-unit reduction in IC score was associated with a 7% increased risk for ADL disability, as well as a 6% increased risk for nursing home admission, and 5% increased risk of mortality over 21 years (24). This is an equivalent increase in the risk of ADL disability to this study but a slightly larger effect for mortality than found in this data (2%). A larger effect for mortality was also described by Locquet et al. (25) who found a 49% decrease in mortality risk with a unit increase in their composite IC score, generated as an average of 4 domain-specific *z*-scores in a Belgian sample of community-dwelling participants. They also found the locomotion and psychological domains in-particular were associated with reduced mortality risk of 55% and 44%, respectively. These variations in the magnitude of the effect may be due to differences between the samples used, with the American and Belgian samples (*n* = 754 and *n* = 481, respectively) being substantially smaller than the ELSA sample, as well as the American participants all taking part in a health plan and Belgian participants recruited mainly from an outpatient clinic and so potentially more health-conscious. It could also be due to variations in the IC score used. Both Stolz et al. (24) and Locquet et al. (25) generated their score by calculating a score for each domain and then taking an average of the domain-specific scores for total IC; although Stolz et al. did compare this to factors scores generated through CFA and found a high correlation between the 2; this may be another reason for different results to this study as 1 unit of IC might reflect a different amount of capacity.

With regards to hospital admission, Yu et al. focused on individual domains of IC and found the cognition and locomotion domains were predictive of visits to emergency departments in a Chinese community-dwelling sample aged ≥60 years, but did not find any domains associated with incident hospitalization in a 1-year follow-up (26). They found a large increase in odds for emergency department visits with cognitive decline (167% increase) and limited mobility (322% increase), which are substantially larger than the magnitude of the effect found in this study. Nevertheless, visits to the emergency department are a different outcome to hospital admissions, as shown in this study, with admissions often reflecting a more serious or ongoing problem that requires more medical intervention. The Yu et al. study also has other methodological differences to the current study, with a smaller sample (*N* = 756) as well as IC measured as individual domain scores based on one indicator binarised to impaired or not impaired. These differences make it difficult to directly compare the results, but it is clear from the current and previous studies that IC, measured using different methods, can predict objective health outcomes in populations around the world, with the cognition and locomotion domains potentially being of key importance, although more evidence would be needed to untangle this relationship.

A key potential difference between the current study and others assessing objective health outcomes is that the mortality and hospital admission information in ELSA is obtained through data linkage for all those who consented to the linkage. This means that ELSA has information on these outcomes even for people who may have later dropped out of the study due to poor health or any other reason, and thus may capture information from people who may not have been included in other studies.

The range of IC models and indicators in the current literature measuring IC with existing data or research studies provides some evidence that the general domains of IC are the important aspect when measuring IC to explore patterns in a population, as opposed to particular tests or indicators. From this study, the item discrimination parameters identified all the locomotion domain indicators and the vitality domain indicator of grip strength as having the highest discrimination and, thus best-mapped individuals along the IC trait continuum. The locomotion domain has been identified in previous research as a predictor of hospital visits as well as mortality, with cognition and psychological domains also showing significant associations with adverse outcomes, suggesting these may be key domains to focus on if all domains cannot be measured. However, differences between the domains were not explicitly tested in this study, with the focus being on testing a total measure of IC that captured the domains of IC and measured all the physical and mental capacities of an individual, as per the WHO framework.

The main strengths of this study include the use of a large nationally representative survey of community-dwelling older adults in England. The ELSA data linkage to health and mortality records allows for objective health outcomes to be examined, with the longitudinal nature of the data meaning an almost 20-year follow-up, which is longer than most other studies of IC’s predictive value. As well as information through linkage, the ELSA survey data provides rich and comprehensive information on the variables of interest and key covariates.

However, there are limitations to this study. Concerning the IC score, the dichotomous indicators are sensitive to the cutoffs; thus, choosing different cutoffs may lead to different results. There are some limitations to the IC indicators. Hearing and vision measurements would be more accurate if assessed with objective tests as opposed to self-reported function. The same could be said for indicators of mobility; however, the inclusion of objective tests of physical function (balance, walking speed, chair rises, and grip strength) in addition to the self-reports of mobility mean that physical function is assessed in a comprehensive manner.

The results of this study have implications for the measurement of IC in research, demonstrating how IRT methodology can be harnessed to create a measure of healthy aging that predicts objective health outcomes in community-dwelling adults. Furthermore, the measures proposed in the model of IC are those commonly collected in biomedical longitudinal population studies. These results contribute to the wider map of IC research, which finds different models of IC also predict key health outcomes for older people, even with different methods and indicators used, beginning to show that the exact replication of indicators and methods may not be crucial to the measurement, but rather the focus on the IC construct and its domains, particularly locomotion. We suggest that the measurement of IC in clinical settings will need to be more controlled and streamlined, as the measures need to be valid, relatively quick to administer, and cover the domains of IC to identify any key areas of decline. The WHO Integrated Care for Older People (ICOPE) framework provides a screening tool for quick IC assessment in clinical settings, which leads to a more in-depth assessment of specific IC domains if declines are seen (27), and this tool is now being used for data collection in the clinical setting and research studies in Europe (28–30).

To conclude, this study finds a novel IRT model of IC to be significantly associated with subsequent functional ability, hospital admissions, and mortality, even when adjusted for socioeconomic and health-related covariates. These results suggest that IC can effectively predict adverse outcomes and potentially identify individuals at risk of functional decline, hospitalization, and death. This has implications for the measurement and monitoring of health in older people and the targeting of interventions ahead of potential adverse health outcomes, supporting the WHO’s focus on IC to promote healthy aging and reduce disability and care dependence.

## Supplementary Material

glac250_suppl_Supplementary_Material

## Data Sharing

The data are freely available and can be accessed through the UK Data Service (SN 8688 and 5050): https://discover.ukdataservice.ac.uk or via the Gateway to Global Aging data: https://g2aging.org/.
