# Supplementary material for: Operationalization of Intrinsic Capacity in Older People and Its Association With Subsequent Disability, Hospital Admission and Mortality: Results From The English Longitudinal Study of Ageing
Source: J Gerontol A Biol Sci Med Sci. 2022 Dec 13;78(4):698–703. doi: 10.1093/gerona/glac250 (PMC10061563; doi:10.1093/gerona/glac250)
Supplement: glac250_suppl_Supplementary_Material [file glac250_suppl_supplementary_material.pdf]

Contents

eTable 1: Intrinsic capacity indicators and cut-offs 2

eTable 2: Covariates at baseline 4

eTable 3: Parameter estimates for the intrinsic capacity indicators from the item response theory model 5

eTable 4: Linear regression between intrinsic capacity scores and sociodemographic and health-related covariates at baseline (fully-adjusted model). Regression coefficients are presented with 95% confidence intervals. (N=4,545) 6

eTable 5: Association between intrinsic capacity scores at baseline and subsequent difficulties with ≥1 ADLs and IADLs after 4 years (N=3,055) and 8 years (N=2,348). Fully adjusted models are presented. 7

eTable 6: Association between intrinsic capacity scores at baseline (as continuous scores and quartiles) and subsequent hospital admission (N=4,489) and mortality (N=4,545) during the 14-year follow-up. Fully-adjusted models are presented. 8

eTable 7: Association between intrinsic capacity and subsequent hospital admission using the Cox proportional hazards model (N=4,489). Fully-adjusted results are presented. 9

eTable 1: Intrinsic capacity indicators and cut-offs

The indicators, their cut-off and the cut-off references are reported, along with the proportion of the sample categorised into "no difficulties" or "difficulties" for each indicator. The total sample N=4,545, although the exact N for each indicator varies due to missing data within individual indicators. Where values of the indicators were missing, imputed values were generated through full information maximum likelihood in the creation of the IC score.

Variable	"No difficulty"		"Difficulty"		Missing	Reference for cut-off
Word recall (20 words, immediate & delayed recall)	Top two tertiles	2,941<br>64.7%	Bottom tertile	1,594<br>35.1%	10<br>0.2%	No accepted cut off.
Orientation (day of the week, day, month, year)	All questions correct	3,578<br>78.7%	≥1 incorrect answer	961<br>21.1%	6<br>0.1%	Low rates of temporal disorientation in older adults (aged >50) (1).
Balance (tandem, semi-tandem and full tandem tests)	Score of 4	3,196<br>70.3%	Score > 4	1,318<br>29.0%	31<br>0.7%	No accepted definition of poor/good performance; 4 is the maximum balance score on the Short Physical Performance Battery (2).
Chair rise test	Five rises within 16.7s	3,265<br>71.8%	Five rises in >16.7s	474<br>10.4%	806<br>17.7%	>16.7s defined as poor performance by the Short Physical Performance Battery (2).
Walking speed	≥0.8 m/s	2,586<br>56.9%	<0.8 m/s	1,626<br>35.8%	333<br>7.3%	Identified as appropriate cut-point for adverse health outcomes by the European Working Group on Sarcopenia in Older People (3,4).
Upper mobility: self-reported difficulties with four actions*	No difficulties	2,933<br>64.5%	≥1 difficulties	1,611<br>35.5%	1<br>0.0%	No accepted cut off.
Lower mobility: self-reported difficulties with six actions**	No difficulties	1,764<br>38.8%	≥1 difficulties	2,780<br>61.2%	1<br>0.0%	No accepted cut off.
Eyesight	Rated good-excellent	3,928<br>86.4%	Rated fair-poor	617<br>13.6%	0<br>0%	Subjective ratings; no accepted cut-off.

Variable	"No difficulty"		"Difficulty"		Missing	Reference for cut-off
Hearing	Rated good-excellent	3,431<br>75.5%	Rated fair-poor	1,114<br>24.5%	0<br>0%	Subjective ratings; no accepted cut-off.
Grip strength	≥30kg (men) or ≥20kg (women)	3,512<br>77.3%	<30kg (men) or <20kg (women)	970<br>21.3%	63<br>1.4%	Low grip strength defined by the European Working Group on Sarcopenia in Older People (3).
Body Mass Index	≥18.5 and <30	3,044<br>67.0%%	<18.5 or ≥30	1,240<br>27.3%	261<br>5.7%	Underweight and obesity identified as thresholds indicating increased risk of mortality and morbidity in older adults. (5,6).
Waist circumference	<94cm (men) or <80cm (women)	935<br>20.6%	≥94cm (men) or ≥80cm (women)	3,460<br>76.1%	150<br>3.3%	Indicator of central obesity in European men and women defined by the International Diabetes Federation (7).
Center for Epidemiology Studies - Depression	Score < 4	3,876<br>85.3%	Score ≥ 4	632<br>13.9%	37<br>0.8%	A score of ≥ 4 is an indicator of depressive symptoms (equivalent to 16 symptom cut-off on 20-item CES-D scale) (8).
Satisfaction With Life Scale	Score ≥ 20	3,850<br>84.7%	Score < 20	656<br>14.4%	39<br>0.9%	≥ 20 represents feelings of neutrality-satisfaction with life as defined by the SWLS (9).

* Reaching/extending arms above shoulder level, pulling or pushing large objects, lifting/carrying weights over 10 pounds (~4.5kg), and picking up a 5-pence coin from a table. ** Walking 100 yards, sitting for two hours, getting up from a chair after a long period of sitting, climbing several flights of stairs without resting, and stooping/kneeling/crouching.

eTable 2: Covariates at baseline

Covariate	Details
Age	Chronological age at time of interview
Sex	Categories: Male, Female
Current legal marital status	Categories: Never married, Married, Separated/Divorced, Widowed
Highest educational qualification	Categories: Degree, A-Level, O-Level or Other qualification, No qualifications
Net total wealth	Quintiles of net total wealth at the benefit unit level. The sum of savings, investments, physical wealth, and housing wealth after financial debt has been subtracted.
Occupation	Question: "Which one of these would you say best describes your current situation?"<br>Categories: Retired/Semi-retired, Employed, Permanently unable to work, Looking after home or family or unemployed
Alcohol consumption	Question: "Thinking now about all kinds of drink, how often have you had an alcoholic drink of any kind in the last 12 months?" Categories: Five or more times per week, Less than five times per week
Smoking status	A derived variable (by the Institute of Fiscal Studies) based on 8 ELSA variables capturing whether someone has ever smoked; uses information from past and present waves. Derivation process outlined in ELSA documentation.<br>Categories: Never smoked, Ex-smoker, Current smoker
Physical activity	A derived variable based on the level of physical activity from respondent's occupation and recreational activities. Derivation process outlined in ELSA documentation.<br>Questions: "Which of these best describes the work that you do in your main job?" & "Do you take part in sports or activities that are vigorous/moderately energetic/mildly energetic? (Frequency)"<br>Derived categories: Sedentary, Low, Moderate, High
Number of chronic health conditions	Count of diagnosed conditions: Alzheimer's disease, angina, arrythmia, arthritis, asthma, cancer, chronic lung disease, coronary heart failure, dementia, diabetes, heart murmur, high blood pressure, high cholesterol, myocardial infarction, osteoporosis, Parkinson's disease, psychiatric problems, stroke.
Self-rated health	Question: "Would you say your health is…?"<br>Categories: Excellent, Very good, Good, Fair, Poor

eTable 3: Parameter estimates for the intrinsic capacity indicators from the item response theory model

Domain	Indicator	Parameters (Standard Error)	
		**Discrimination**	**Difficulty**
**Cognition**	Word recall	0.690 (0.040)	-0.748 (0.058)
	Orientation	0.373 (0.040)	-3.336 (0.347)
**Locomotion**	Chair rises	1.432 (0.088)	-1.478 (0.075)
	Balance test	1.325 (0.058)	-0.759 (0.036)
	Walking speed	1.826 (0.082)	-0.235 (0.025)
	Lower mobility	1.956 (0.090)	0.412 (0.025)
	Upper mobility	2.255 (0.100)	-0.417 (0.023)
**Sensory**	Eyesight	1.071 (0.058)	-1.915 (0.084)
	Hearing	0.576 (0.041)	-2.059 (0.141)
**Vitality**	Grip strength	1.469 (0.066)	-1.077 (0.041)
	BMI	0.462 (0.041)	-1.967 (0.178)
	Waist circumference	0.323 (0.043)	4.210 (0.544)
**Psychological wellbeing**	CES-D	1.182 (0.062)	-1.802 (0.074)
	Satisfaction With Life Scale	0.854 (0.057)	-2.280 (0.134)

eTable 4: Linear regression between intrinsic capacity scores and sociodemographic and health-related covariates at baseline (fully adjusted model). Regression coefficients are presented with 95% confidence intervals. (N=4,545)

Predictors		Coefficients [95% CI]
**Age (years)**		-0.32** [-0.35, -0.30]
**Sex**<br>Ref = Male	Female	-2.90** [-3.33, -2.48]
**Marital status**	Never married	-0.74 [-1.69, 0.22]
Ref = Married	Separated/divorced	-0.42 [-1.17, 0.32]
	Widowed	-0.81* [-1.35, -0.28]
**Education**	A-Level	0.06 [-0.71, 0.82]
Ref = Degree	O-Level/Other	-0.36 [-1.08, 0.36]
	None	-1.06* [-1.79, -0.33]
**Wealth quintile**	1 (Lowest)	-3.16** [-3.88, -2.43]
Ref = Highest	2	-2.28** [-2.94, -1.62]
	3	-1.29** [-1.91, -0.67]
	4	-1.04* [-1.64, -0.44]
**Occupation**	Retired/Semi-retired	-0.86* [-1.49, -0.23]
Ref = Employed	Permanently unable to work	-5.63** [-6.94, -4.33]
	Looking after home/family or unemployed	-1.00* [-1.85, -0.15]
**Smoking status**	Ex-smoker	-0.83** [-1.26, -0.40]
Ref = Never smoked	Current smoker	-0.56 [-1.23, 0.10]
**Alcohol consumption**<br>Ref = <5 days a week	≥5 days week	0.60* [0.13, 1.07]
**Physical activity**	Sedentary	-5.29** [-6.20, -4.39]
Ref = Moderate	Low	-2.75** [-3.23, -2.26]
	High	1.43** [0.88, 1.98]
**No. of health conditions**		-0.36** [-0.53, -0.19]
**Self-rated health**	Very good	-1.59** [-2.27, -0.92]
Ref = Excellent	Good	-5.06** [-5.73, -4.39]
	Fair	-9.37** [-10.13, -8.62]
	Poor	-12.49** [-13.52, -11.46]

*p<0.05 **p<0.001

eTable 5: Association between intrinsic capacity scores at baseline and subsequent difficulties with ≥1 ADLs and IADLs after 4 years (N=3,055) and 8 years (N=2,348). Fully adjusted models are presented.

**Predictors**		**ADLs (4 years) OR [95% CI]**	**ADLs (8 years) OR [95% CI]**	**IADLs (4 years) OR [95% CI]**	**1 IADLs (8 years) OR [95% CI]**
**Intrinsic Capacity score**		0.93** [0.91, 0.94]	0.93** [0.91, 0.95]	0.90** [0.89, 0.92]	0.92** [0.91, 0.94]
**Age (years)**		1.04** [1.02, 1.06]	1.04** [1.02, 1.07]	1.05** [1.03, 1.07]	1.06** [1.04, 1.08]
**Sex** Ref = Male	Female	0.82 [0.64, 1.04]	0.94 [0.72, 1.22]	1.53** [1.21, 1.94]	1.13 [0.87, 1.46]
**Baseline (I)ADLs difficulties** Ref = None	Difficulties with ≥1	4.40** [3.46, 5.58]	3.61** [2.75, 4.75]	1.83** [1.43, 2.33]	1.94** [1.47, 2.56]
**Marital**	Never married	1.82* [1.13, 2.94]	1.24 [0.71, 2.16]	0.96 [0.59, 1.59]	0.79 [0.44, 1.43]
**status**	Separated/divorced	1.26 [0.86, 1.84]	1.33 [0.89, 1.99]	0.73 [0.49, 1.09]	1.12 [0.75, 1.66]
Ref = Married	Widowed	0.90 [0.68, 1.20]	1.04 [0.76, 1.44]	1.12 [0.86, 1.45]	1.16 [0.85, 1.57]
**Education**	A-Level	1.10 [0.73, 1.67]	1.11 [0.71, 1.74]	1.03 [0.68, 1.56]	1.22 [0.79, 1.90]
Ref = Degree	O-Level/Other	0.93 [0.62, 1.37]	1.06 [0.69, 1.62]	0.80 [0.54, 1.18]	0.84 [0.55, 1.29]
	None	1.03 [0.70, 1.53]	1.25 [0.81, 1.93]	0.90 [0.61, 1.33]	1.09 [0.71, 1.67]
**Wealth quintile**	1 (Lowest)	1.01 [0.69, 1.49]	0.79 [0.51, 1.23]	0.87 [0.59, 1.29]	1.06 [0.69, 1.63]
Ref = Highest	2	0.88 [0.62, 1.25]	0.99 [0.67, 1.46]	1.03 [0.73, 1.46]	1.49* [1.02, 2.18]
	3	0.83 [0.59, 1.17]	0.89 [0.62, 1.29]	1.24 [0.89, 1.73]	1.18 [0.82, 1.71]
	4	0.86 [0.62, 1.20]	1.01 [0.71, 1.44]	1.20 [0.87, 1.65]	1.30 [0.92, 1.84]
**Occupation**	Retired/Semi-retired	0.70 [0.48, 1.01]	0.91 [0.62, 1.35]	0.95 [0.64, 1.40]	0.81 [0.56, 1.19]
Ref = Employed	Permanently unable to work	1.01 [0.52, 1.96]	1.07 [0.51, 2.22]	1.18 [0.61, 2.26]	1.30 [0.63, 2.70]
	Looking after home/family or unemployed	0.78 [0.48, 1.26]	0.74 [0.44, 1.24]	0.79 [0.49, 1.29]	0.89 [0.54, 1.46]
**Smoking status**	Ex-smoker	1.02 [0.81, 1.28]	1.08 [0.84, 1.39]	0.89 [0.71, 1.11]	0.92 [0.72, 1.17]
Ref = Never smoked	Current smoker	1.11 [0.78, 1.58]	1.04 [0.70, 1.57]	1.32 [0.94, 1.86]	1.21 [0.82, 1.77]
**Alcohol consumption** Ref = <5 days a week	≥5 days week	0.91 [0.70, 1.19]	1.30 [0.97, 1.74]	1.11 [0.86, 1.43]	1.22 [0.91, 1.62]
**Physical activity**	Sedentary	1.91* [1.11, 3.28]	2.09* [1.02, 4.25]	1.00 [0.59, 1.69]	1.54 [0.77, 3.11]
Ref = Moderate	Low	1.03 [0.80, 1.32]	1.11 [0.84, 1.46]	0.95 [0.75, 1.20]	1.09 [0.83, 1.41]
	High	0.95 [0.69, 1.32]	1.01 [0.73, 1.41]	0.96 [0.70, 1.33]	0.76 [0.55, 1.06]
**No. of health conditions**		1.06 [0.97, 1.16]	1.00 [0.90, 1.10]	1.06 [0.97, 1.15]	1.11* [1.01, 1.22]
**Self-rated health**	Very good	1.56 [0.95, 2.54]	2.15* [1.25, 3.70]	1.04 [0.66, 1.63]	1.55 [0.96, 2.52]
Ref = Excellent	Good	1.97* [1.22, 3.18]	2.88** [1.69, 4.90]	1.63* [1.05, 2.52]	1.86* [1.16, 2.98]
	Fair	2.75** [1.65, 4.60]	3.40** [1.91, 6.05]	2.47** [1.54, 3.96]	2.70** [1.62, 4.52]
	Poor	3.73** [1.96, 7.09]	4.06** [1.96, 8.43]	4.19** [2.28, 7.69]	3.75** [1.89, 7.44]

OR = odds ratio; *p<0.05 **p<0.001

eTable 6: Association between intrinsic capacity scores at baseline (as continuous scores and quartiles) and subsequent hospital admission (N=4,489) and mortality (N=4,545) during the 14-year follow-up. Fully adjusted models are presented.

**Predictors**		**Hospital admission SHR [95% CI]**	**Mortality HR [95% CI]**
**Intrinsic capacity score (continuous)**		0.99** [0.98, 0.99]	0.98** [0.98, 0.99]
**Intrinsic capacity score**	1 (Lowest)	1.32** [1.16, 1.51]	1.46** [1.21, 1.77]
**(quartiles)**	2	1.28** [1.15, 1.41]	1.30* [1.10, 1.54]
Ref = Highest	3	1.08 [0.99, 1.18]	1.15 [0.97, 1.35]
**Age (years)**		1.02** [1.01, 1.02]	1.10** [1.09, 1.11]
**Sex**	Female		
Ref = Male		0.93 [0.86, 1.00]	0.56** [0.50, 0.62]
**Marital**	Never married	0.98 [0.83, 1.15]	1.25 [1.01, 1.56]
**status**	Separated/divorced	1.08 [0.95, 1.22]	1.08 [0.89, 1.32]
Ref = Married	Widowed	0.92 [0.84, 1.01]	1.17* [1.04, 1.31]
**Education**	A-Level	1.09 [0.95, 1.24]	1.00 [0.81, 1.23]
Ref = Degree	O-Level/Other	0.99 [0.87, 1.12]	1.00 [0.83, 1.22]
	None	0.98 [0.86, 1.12]	1.03 [0.85, 1.26]
**Wealth quintile**	1 (Lowest)	1.14 [1.00, 1.29]	1.05 [0.88, 1.26]
Ref = Highest	2	1.21* [1.08, 1.35]	0.99 [0.83, 1.17]
	3	1.04 [0.93, 1.15]	1.08 [0.91, 1.27]
	4	1.08 [0.98, 1.19]	1.07 [0.91, 1.25]
**Occupation**	Retired/Semi-retired	1.04 [0.94, 1.16]	1.23 [0.98, 1.55]
Ref = Employed	Permanently unable to work	1.08 [0.83, 1.40]	1.45* [1.04, 2.00]
	Looking after home/family or unemployed	0.99 [0.86, 1.15]	1.09 [0.82, 1.43]
**Smoking status**	Ex-smoker	1.01 [0.93, 1.08]	1.17* [1.05, 1.30]
Ref = Never smoked	Current smoker	1.02 [0.92, 1.14]	1.93** [1.66, 2.26]
**Alcohol consumption**	≥5 days week		
Ref = <5 days a week		1.05 [0.97, 1.14]	0.96 [0.86, 1.09]
**Physical activity**	Sedentary	1.00 [0.84, 1.19]	1.42** [1.19, 1.68]
Ref = Moderate	Low	1.04 [0.96, 1.13]	1.14* [1.02, 1.28]
	0.99 [0.90, 1.08]	0.91 [0.78, 1.07]	0.91 [0.78, 1.07]
**No. of health conditions**		1.09** [1.06, 1.13]	1.06* [1.02, 1.10]
**Self-rated health**	Very good	1.17* [1.05, 1.31]	1.28* [1.03, 1.59]
Ref = Excellent	Good	1.22* [1.09, 1.37]	1.49** [1.20, 1.84]
	Fair	1.43** [1.25, 1.65]	1.81** [1.44, 2.28]
	Poor	1.36* [1.09, 1.68]	2.38** [1.82, 3.11]

SHR = subdistribution hazard ratio; HR = hazard ratio; *p<0.05 **p<0.001

eTable 7: Association between intrinsic capacity and subsequent hospital admission using the Cox proportional hazards model (N=4,489). Fully adjusted results are presented.

**Predictors**		**Hospital admission HR [95% CI]**
**Intrinsic capacity score**		0.99** [0.98, 0.99]
**Age (years)**		1.02** [1.01, 1.02]
**Sex** Ref = Male	Female	0.93* [0.86, 1.00]
**Marital status**	Never married	0.98 [0.83, 1.15]
	Separated/divorced	1.07 [0.95, 1.22]
Ref = Married	Widowed	0.92 [0.84, 1.01]
**Education**	A-Level	1.09 [0.96, 1.24]
Ref = Degree	O-Level/Other	0.99 [0.87, 1.12]
	None	0.98 [0.87, 1.11]
**Wealth quintile**	1 (Lowest)	1.14* [1.00, 1.29]
Ref = Highest	2	1.21* [1.08, 1.35]
	3	1.04 [0.93, 1.15]
	4	1.08 [0.97, 1.20]
**Occupation**	Retired/Semi-retired	1.05 [0.94, 1.17]
Ref = Employed	Permanently unable to work	1.08 [0.87, 1.35]
	Looking after home/family or unemployed	0.99 [0.86, 1.15]
**Smoking status**	Ex-smoker	1.01 [0.94, 1.08]
Ref = Never smoked	Current smoker	1.02 [0.91, 1.14]
**Alcohol consumption** Ref = <5 days a week	≥5 days week	1.05 [0.97, 1.13]
**Physical activity**	Sedentary	1.00 [0.86, 1.16]
Ref = Moderate	Low	1.04 [0.96, 1.13]
	High	0.99 [0.90, 1.08]
**No. of health conditions**		1.09** [1.06, 1.12]
**Self-rated health**	Very good	1.17* [1.04, 1.32]
Ref = Excellent	Good	1.22* [1.08, 1.38]
	Fair	1.43** [1.25, 1.64]
	Poor	1.36* [1.12, 1.63]

HR = hazard ratio; *p<0.05 **p<0.001

References

1. Benton AL, Eslinger PJ, Damasio AR. Normative observations on neuropsychological test performances in old age. *Journal of Clinical Neuropsychology*. 1981;3(1):33-42. doi:https://doi.org/10.1080/01688638108403111
2. Guralnik JM, Ferrucci L, Pieper CF, et al. Lower Extremity Function and Subsequent Disability: Consistency Across Studies, Predictive Models, and Value of Gait Speed Alone Compared With the Short Physical Performance Battery. *The Journals of Gerontology Series A: Biological Sciences and Medical Sciences*. 2000;55(4):M221-M231. doi:https://doi.org/10.1093/gerona/55.4.m221
3. Cruz-Jentoft AJ, Baeyens JP, Bauer JM, et al. Sarcopenia: European consensus on definition and diagnosis: Report of the European Working Group on Sarcopenia in Older People. *Age and Ageing*. 2010;39(4):412-423. doi:https://doi.org/10.1093/ageing/afq034
4. Abellan Van Kan G, Rolland Y, Andrieu S, et al. Gait speed at usual pace as a predictor of adverse outcomes in community-dwelling older people an International Academy on Nutrition and Aging (IANA) Task Force. *The journal of nutrition, health & aging*. 2009;13(10):881-889. doi:https://doi.org/10.1007/s12603-009-0246-z
5. Mathus-Vliegen EMH, Basdevant A, Finer N, et al. Prevalence, Pathophysiology, Health Consequences and Treatment Options of Obesity in the Elderly: A Guideline. *Obesity Facts*. 2012;5(3):460-483. doi:https://doi.org/10.1159/000341193
6. Charlton KE, Batterham MJ, Bowden S, et al. A high prevalence of malnutrition in acute geriatric patients predicts adverse clinical outcomes and mortality within 12 months. *e-SPEN Journal*. 2013;8(3):e120-e125. doi:https://doi.org/10.1016/j.clnme.2013.03.004
7. Alberti KGMM, Zimmet P, Shaw J. International Diabetes Federation: a consensus on Type 2 diabetes prevention. *Diabetic Medicine*. 2007-05-01 2007;24(5):451-463. doi:https://doi.org/10.1111/j.1464-5491.2007.02157.x
8. Radloff LS. The CES-D Scale: A Self-Report Depression Scale for Research in the General Population. *Applied Psychological Measurement*. 1977;1(3):385-401. doi:https://doi.org/10.1177/014662167700100306
9. Pavot W, Diener E. The Satisfaction With Life Scale and the emerging construct of life satisfaction. *The Journal of Positive Psychology*. 2008-04-01 2008;3(2):137-152. doi:https://doi.org/10.1080/17439760701756946
